# Comparison of conventional ultrasonography and elastography ultrasound in detecting malignant cervical lymph nodes

**DOI:** 10.12669/pjms.41.6.9215

**Published:** 2025-06

**Authors:** Jun Peng Zhang, Yan Fei Xia, Xue Han, Lin Shi

**Affiliations:** 1 Jun Peng Zhang, Department of Ultrasonography, Shandong Provincial Hospital affiliated, Shandong First Medical University, Jinan, Shandong, 250021, China; 2 Yan Fei Xia, Department of Ultrasonography, Shandong Provincial Hospital affiliated, Shandong First Medical University, Jinan, Shandong, 250021, China; 3 Xue Han, Department of Ultrasonography, Shandong Provincial Hospital affiliated, Shandong First Medical University, Jinan, Shandong, 250021, China; 4 Lin Shi, Department of Ultrasonography, Shandong Provincial Hospital affiliated, Shandong First Medical University, Jinan, Shandong, 250021, China

**Keywords:** Ultrasonography, Strain elastography, Virtual touch tissue imaging quantification, Cervical lymph nodes

## Abstract

**Background and Objective:**

In the contemporary landscape, conventional ultrasonography (US) stands as the primary modality for diagnosing cervical lymphadenopathy. While ultrasound elastography introduces an avenue for discerning the tissue hardness of lymph nodes (LNs) and contributes additional evidence for a conclusive diagnosis, skepticism persists among clinicians regarding its routine diagnostic utility, primarily attributed to the restricted case inclusion prevalent in most published reports. This study aimed to compare the performance of strain elastography (SE) and Virtual Touch Tissue Imaging Quantification (VTIQ) with that of conventional US for the measurement of cervical LNs.

**Methods:**

We performed a retrospective chart review of patients with cervical lymphadenopathy who underwent both conventional ultrasound (US) and elastography ultrasound at Shandong Provincial Hospital affiliated to Shandong First Medical University from January 2021 to December 2022.The stiffness of 123 abnormal cervical lymph nodes (LNs) in 106 patients, substantiated by pathologic diagnoses, was quantified through elastography. Conventional US, strain elastography (SE), and Virtual Touch Tissue Imaging Quantification (VTIQ) were employed for the comprehensive evaluation of all identified LNs. The diagnostic efficacy of elastography’s quantitative analysis was meticulously assessed utilizing receiver operating characteristic (ROC) curves.

**Results:**

Pathological analysis affirmed malignancy in 89 out of 123 abnormal cervical lymph nodes (LNs) within the cohort of 106 patients. The area under the ROC curve (AUC) revealed a significantly greater discriminatory capacity for malignant LNs with Virtual Touch Tissue Imaging Quantification (VTIQ) compared to Strain Elastography (SE) (AUC, 0.84 vs. 0.74; z=2.83, p=0.005). VTIQ exhibited superior specificity, false-negative ratio, and false-positive ratio in contrast to SE. However, SE demonstrated heightened sensitivity in the detection of malignant LNs.

**Conclusions:**

Both Strain Elastography (SE) and Virtual Touch Tissue Imaging Quantification (VTIQ) demonstrated commendable diagnostic performance for malignant nodes, with VTIQ exhibiting superior conspicuity over SE. The amalgamation of conventional ultrasonography with elastography enhances the precision of ultrasound diagnosis in cervical lymph node diseases.

## INTRODUCTION

The precise diagnosis of lymph nodes is of paramount importance in formulating effective clinical treatment strategies for patients. Conventional ultrasonography (US), as the preferred imaging modality, offers real-time, convenient, and non-invasive examination capabilities, distinguishing itself from computed tomography (CT) and magnetic resonance imaging (MRI). The integration of US-guided fine needle aspiration biopsy further enhances diagnostic specificity to an impressive 93%.^1^-^3^ Positioned as a secure and noninvasive technique, ultrasound presents the dual advantages of cost-effectiveness and minimal complications. However, the clinical accuracy of conventional US remains suboptimal, characterized by low specificity in discriminating lymph nodes.^4^ In response to these challenges, ultrasound elastography technologies, exemplified by strain elastography (SE) imaging and Virtual Touch Tissue Imaging Quantification (VTIQ), have emerged. These advancements aim to noninvasively estimate the elastic modulus of tissues, offering novel avenues for lymph node diagnosis.^5^,^6^

Cervical lymph node abnormalities encompass a spectrum of conditions such as reactive hyperplasia, lymphoma, lymphadenitis, granuloma, metastasis, and tuberculosis. While select studies have reported favorable outcomes in detecting malignant cervical lymph nodes using quantitative elasticity technology,^7^,^8^ the application of elastosonography techniques for lymphadenopathy diagnosis remains a topic of controversy. Moreover, scant attention has been devoted to investigating the divergences in the diagnostic performance of widely employed elastosonography techniques for lymphadenopathy. The potential for disparate outcomes with different elastosonography methods within the same subject raises concerns among operators.^9^,^10^ Utilizing histological findings as a reference, our investigation delved into lymph node (LN) stiffness values to delineate between malignant and benign LNs. This exploration concurrently assessed and juxtaposed the diagnostic efficacy of Virtual Touch Tissue Imaging Quantification (VTIQ) and Strain Elastography (SE) in discerning malignant LNs.

## METHODS

This was a retrospective study. Inclusion criteria comprised patients meeting predefined standards and possessing abnormal cervical lymph nodes (LNs) identified through ultrasound at our hospital. Exclusion criteria encompassed individuals who had undergone specific treatment or cervical surgery preceding the examination. From January 2021 to December 2022, a total of 123 consecutive abnormal cervical LNs in 106 patients, who underwent both conventional ultrasound (US) and elastography ultrasound, were included.

### Ethical Approval:

The study was approved by the Institutional Ethics Committee of Shandong Provincial Hospital affiliated to Shandong First Medical University on July 15, 2024 (No. SWYX2024-379), and written informed consent was obtained from all participants.

Sonographic characteristics of the abnormal LNs were systematically documented for all enrolled patients. The conclusive determination of malignant LNs was contingent upon histopathological examination results. All ultrasound examinations were meticulously scrutinized by a seasoned radiologist with over five years of expertise, ensuring consistency in interpretation. The Ultrasonography Department’s team of radiologists conducted the examinations. A US system, either the Hitachi Preirus (7.5-13.0 MHz) or Acuson S3000 (4-9 MHz), facilitated the conventional US scans. In instances where multiple abnormal lymph nodes (LNs) were present, the largest among them was designated as the index lesion for evaluation. Documented parameters encompassed the count, dimensions, morphology, echogenicity, and vascular distribution for each examination.

The SE procedure employed 7.5-13.0 MHz linear array probes through the Hitachi Preirus, with patients positioned in the supine orientation. Utilizing conventional ultrasonography (US) as a guide, SE was implemented. During LN stiffness measurement, patients observed a five seconds breath hold and were instructed to refrain from swallowing during inspiration. The quantitative elastography technique involved applying gentle, repetitive compression on the skin above the LN to induce tissue deformation, necessitating careful avoidance of bones or blood vessels within the scanning area. The sampling frame encompassed both the cervical lymph node and surrounding neck muscles. Optimal images were selected and saved for subsequent evaluation. The integrated system calculated the AREA% of the largest rectangle delineated within the LNs by the radiologist. A minimum of five calculations were documented, and the mean value was utilized for quantitative analysis.

The patient maintained the same position during the VTIQ examination as in the Strain Elastography (SE) procedure. Employing the Acuson S3000 linear array transducer (4-9 MHz), a low-frequency longitudinal push pulse facilitated the measurement. The device gauged the speed of shear waves (perpendicular to the longitudinal wave) through a detection pulse. Simultaneously, manual compression was minimized to prevent tissue stiffening, ensuring optimal reproducibility of the image. Within the VTIQ image, the evaluation of quality, time, displacement, and velocity patterns informed the determination of tissue stiffness. The static and quality graphs, featuring green for good quality, yellow denoting an edge state, and red indicating poor quality, played a pivotal role. Shear wave velocity (SWV) within the region of interest (ROI) was quantitatively measured in meters per second (m/s), capped at 10 m/s. Our analysis selected five to six points within the ROI, with the VTIQ value representing the mean of these points.

### Statistical analysis:

It was performed using SPSS 28.0 (IBM Corp., Armonk, NY, USA). With quantitative data presented as the mean ± standard deviation. Categorical variables were compared using the chi-square test, while group comparisons utilized One-Way ANOVA. The receiver operating characteristic (ROC) curve, with the final histopathological diagnosis as the reference standard, was employed to analyze stiffness measurements. The optimal cutoff value, maximizing the sum of sensitivity and specificity, was identified to compare diagnostic sensitivities, specificities, and accuracies between the two elastography methods, utilizing the McNemar test. A significance threshold of *p*<0.05 was established for statistical significance.

## RESULTS

The final analysis encompassed 123 lymph nodes (LNs) from 106 patients. Histologic examination delineated 34 benign LNs, comprising 21 cases of nonspecific lymphadenitis and 13 cases of tuberculous origin. The remaining 89 LNs were malignant, with a breakdown of 43 adenocarcinomas,22 squamous carcinomas,15 papillary thyroid carcinomas, and nine small cell carcinomas. The study group included 45 men (mean age: 49.6 ± 14.6 years, range: 17–77 years) and 61 women (mean age: 49.6 ± 14.6 years, range: 17–77 years). The mean age of patients with malignant LNs (53.5 ± 12.3 years) was significantly higher than that of patients with benign LNs (43.1 ± 15.9 years).

No gender-based differences were observed between patients with benign or malignant LNs. Table-I delineates various conventional US features, revealing notable distinctions between benign and malignant LNs. The resistance index (RI), long to short axis ratio (L/S ratio), and the absence of an echogenic hilum demonstrated significant differences. Malignant LNs exhibited a significantly higher RI compared to benign LNs. Conventional US findings for malignant LNs included a consistent round or petal shape, diminished L/S ratio, and the absence of a hilum. No statistically significant variations were observed in the number of LNs and tissue necrosis within each patient between the two groups.

**Table-I:** Ultrasonography characteristics of 123 cervical LNs.

Characteristics	Malignant LNs	Benign LNs	P value
Single/multiple	17/72	3/31	0.273
Absent hilum (Y/N)	11/78	13/21	0.002
Necrosis (Y/N)	18/71	11/23	0.656
L/S ratio (≥2/<2)	13/76	16/18	0.000
RI (≤0.7/>0.7)	38/51	28/6	0.000
AREA% (≤45.23/>45.23)	16/73	23/11	0.000
SWV (≤3.05/>3.05)	7/82	28/6	0.000

**Fig.1:**
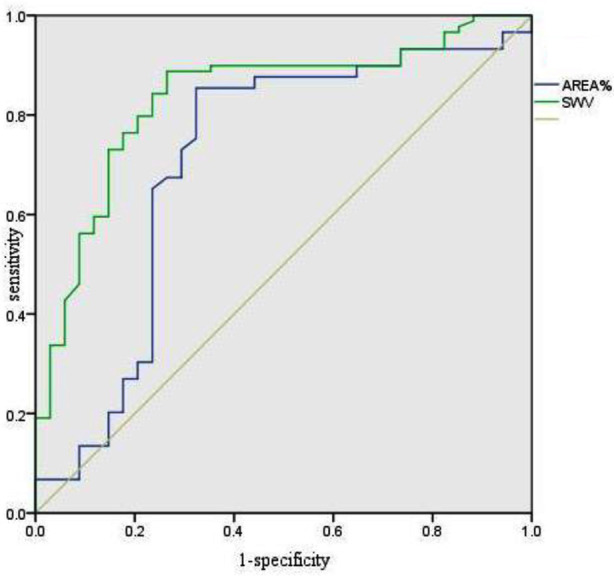
Receiver operating characteristic curve for AREA% and SWV.

Significant disparities were observed in the AREA% (Strain Elastography - SE) and shear wave velocity (SWV - Virtual Touch Tissue Imaging Quantification - VTIQ) values between malignant LNs (58.39±18.64% and 4.39±1.24 m/s) and benign LNs (43.26±18.39% and 2.89±0.88 m/s) (*p*=0.000). Receiver operating characteristic (ROC) curves were constructed for AREA% and SWV to delineate the distinction between malignant and benign nodes. An AREA% cutoff value of 45.23% exhibited a sensitivity of 84.3% and specificity of 70.6%. For SWV, the optimal cutoff value of 3.05 m/s yielded a sensitivity of 88.8% and specificity of 73.5%.ROC analysis highlighted a significantly larger area under the ROC curve (AUC) for VTIQ (0.84) compared to SE (0.74) (z=2.83, *p*=0.005) (Table-II, Fig.1).

**Table-II:** Performance Characteristics of CUS and elastography US.

	Pathology		Performance Characteristics						
	Malignant	Benign	Cutoff	Sensitivity, %	Specificity, %	PPV, %	NPV, %	P value
Absent hilum (Y/N)	11/78	13/21	Absent hilum	87.6	38.2	78.8	54.2	0.002
L/Sratio (≥2/<2)	13/76	16/18	<2	85.4	47.1	80.9	55.2	0.000
RI (≤0.7/>0.7)	38/51	28/6	>0.7	57.3	82.4	89.5	42.4	0.000
AREA% (≤45.23/>45.23)	16/73	23/11	45.23%	82.0	67.6	86.9	93.2	0.000
SWV (≤3.05/>3.05)	7/82	28/6	3.05	92.1	82.4	93.2	80.0	0.000

Within our study, the SE imaging set exhibited 11 false-positive nodes, while the VTIQ imaging set had nine false-positive nodes. False-negative findings were noted in 13 LNs with SE and 10 LNs with VTIQ (Table-III). The chi-square test indicated that VTIQ held an advantage over SE in terms of both the misdiagnosis rate and the missed diagnosis rate. However, no statistically significant differences were observed in the sensitivity and specificity of the two elastography techniques at the 5% level (*p*=0.06; *p*=0.22).

**Table-III:** Comparison between SE and VTIQ.

		Malignant LN			Benign LN		
		SE			SE		
		+	-	Total	+	-	Total
VTIQ	+	70	9	79	8	1	9
	-	6	4	10	3	22	25
	Total	76	13	89	11	23	34

## DISCUSSION

Numerous studies have investigated the efficacy of conventional ultrasound in the diagnosis of lymphadenopathy. Reports show that its sensitivity ranges from 26% to 76% and its specificity ranges from 44% to 98%.^11^-^15^ Conventional ultrasound has advantages in depicting the characteristics of cervical lymph nodes with different morphological changes. These characteristics include factors such as quantity, long - to - short axis ratio (L/S ratio), lymph node hilum, necrosis, and resistance index (RI). Conventional ultrasound remains an important method for diagnosing lymph node diseases and has high diagnostic accuracy, which is reflected in many studies.^7^,^16^ In our study, there were significant differences between benign and malignant cervical lymph nodes in terms of resistance index, L/S ratio, and the presence of hyperechoic lymph node hilum.

**Fig.2:**
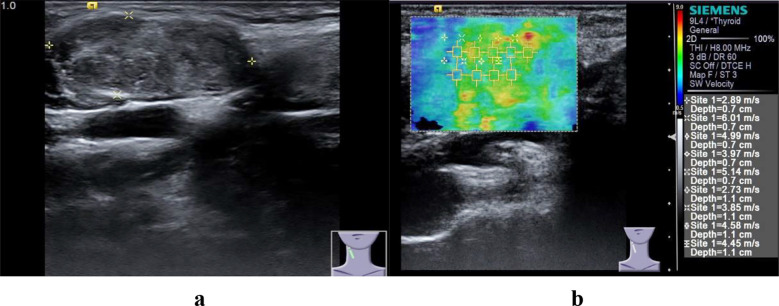
Malignant cervical LN in a 64-year-old woman. a, Conventional ultrasonic images of malignant LN. b, Elastic ultrasound image of malignant LN.

**Fig.3:**
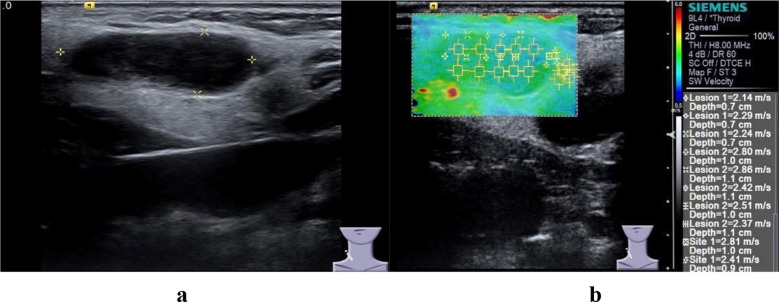
Benign cervical LN in a 34-year-old woman. a, Conventional ultrasonic images of malignant LN. b, Elastic ultrasound image of malignant LN.

In elastic ultrasound, the hardness values of malignant lymph nodes (Fig.2 and 3) are higher than those of benign lymph nodes, and this research result has been confirmed in many studies.^16^,^17^ At the same time, studies have shown that elastic ultrasound exhibits high sensitivity and specificity in the diagnosis of benign and malignant lymph nodes. The difference in the hardness of lymph nodes is attributed to the pathological changes inside the lymph nodes. There are tumor cell infiltration, necrosis or calcification inside malignant lymph nodes, and this pathological change is different from that of benign hyperplastic lymph nodes.

Lymph node tuberculosis is prone to necrosis and calcification. This pathological change will lead to an increase in the hardness of lymph node tissue. The research of Wang J,et al confirms this view.^18^ In our study, in the strain elastography (SE) imaging group, 11 false - positive lymph nodes (LNs) were found, and in the virtual touch tissue imaging quantification (VTIQ) imaging group, 9 false - positive lymph nodes were found. The false - positive lymph nodes in both groups were tuberculous lymph nodes, which also confirmed this conclusion. Local infiltration of malignant tumor cells in the lymph nodes/local necrosis in the lymph nodes can affect the overall hardness of the lymph nodes. In these cases, the overall hardness of the lymph nodes will not change significantly, thus affecting the diagnostic results.

This conclusion was confirmed in our study. In the false - negative cases in the diagnosis of the two techniques, there was local tumor cell infiltration/necrosis in multiple lymph nodes. At the same time, we found that 4 lymph nodes were misdiagnosed by both elastic ultrasound techniques. The diameters of these 4 lymph nodes were all less than 5 mm, and there was no necrosis. This finding is consistent with the research results of Cetin Tuncez H et al.^19^ All false-positive lymph nodes in the SE imaging group and two false-positive lymph nodes in the VTIQ imaging group were related to lymph node calcification without necrosis. In addition, other misdiagnosed lymph nodes in the VTIQ imaging group may have been affected by motion artifacts.

The number of false-negative and false-positive lymph nodes in the VTIQ imaging group was less than that in the SE imaging group. This difference may be attributed to the fact that VTIQ can select multiple points, thus avoiding the influence of coarse calcification and necrosis on the results. Despite these differences, no significant differences were found in the diagnostic performance of the two elastography methods in terms of sensitivity and specificity. However, the diagnostic performance of virtual touch tissue imaging quantification (VTIQ) was relatively higher than that of strain elastography (SE). Our research results have confirmed that the tissue hardness value can serve as a valuable predictive indicator for the elastographic ultrasound diagnosis of abnormal lymph nodes (LN).

In simple data statistical analysis, the ROC curve shows the optimal cut - off values of AREA% and SWV. When differentiating between benign and malignant lymph nodes (LN), their sensitivities are 84.3% and 88.8% respectively, and their specificities are 70.6% and 73.5% respectively. The area under the curve (AUC) of SWV (0.84) is significantly larger than that of AREA% (0.74). The data indicate that virtual touch tissue imaging quantification (VTIQ) has the potential to improve the accuracy of the differential diagnosis of malignant and benign lymph nodes, even in cases involving necrotic, calcified or small - sized lymph nodes. Based on this, we believe that using VTIQ is more likely to avoid misclassification of diseased lymph nodes. The same view was confirmed in the study by Cetin Tuncez H et al.^19^

In the ultrasonic diagnostic research of lymph node diseases, conventional ultrasound remains the basis and an important diagnostic method. Elastography is a diagnostic method superimposed on conventional ultrasound and should not be used in isolation from conventional ultrasound examinations. Some studies show that elastography cannot increase the diagnostic accuracy of lymph node diseases. However, in our study, elastography can provide additional diagnostic information in the diagnosis of lymph node diseases and improve the doctor’s diagnostic confidence.

### Limitation

However, it’s crucial to acknowledge certain limitations in our study. Firstly, lymphoma was excluded, given its potential to exhibit softer characteristics than metastatic LNs from other malignancies. Additionally, the study did not document interobserver variability, representing a potential area for future investigation and refinement.

## CONCLUSION

In summary, when considering a singular index, elastography ultrasound outperforms conventional ultrasound in both sensitivity and specificity. However, in the broader context of long-term clinical practice, conventional ultrasound continues to serve as the gold standard for lymph node (LN) diagnosis, offering a more comprehensive array of information for disease diagnosis.^24^ Elastosonography, as an innovative ultrasound technology, contributes valuable insights into LN stiffness, thereby enhancing diagnostic confidence. Our study underscores that Virtual Touch Tissue Imaging Quantification (VTIQ) exhibits superior diagnostic performance compared to Strain Elastography (SE) in the context of abnormal LNs.

### Authors’ Contribution:

**JZ** and **LS:** Conceived, designed and did statistical analysis & editing of manuscript, is responsible for integrity of research.

**YX** and **XH**: Literature search, Did data collection and manuscript writing.

**LS:** Critical review and final approval of manuscript.

All authors have read and approved the final manuscript.
